# Genome-wide association study of rust traits in orchardgrass using SLAF-seq technology

**DOI:** 10.1186/s41065-017-0027-3

**Published:** 2017-02-23

**Authors:** Bing Zeng, Haidong Yan, Xinchun Liu, Wenjing Zang, Ailing Zhang, Sifan Zhou, Linkai Huang, Jinping Liu

**Affiliations:** 1 Department of Animal Science, Southwest University, Rongchang, Chongqing, 402460 China; 2 Department of Grassland Science, Animal Science and Technology College, Sichuan Agricultural University, Chengdu, 611130 China; 3 Agricultural College, Sichuan Agricultural University, Chengdu, 611130 China; 4 College of Life Science, China West Normal University, Nanchong, 637009 China

**Keywords:** Genome-wide association study, Orchardgrass, Rust disease, SLAF-seq

## Abstract

**Background:**

While orchardgrass (*Dactylis glomerata* L.) is a well-known perennial forage species, rust diseases cause serious reductions in the yield and quality of orchardgrass; however, genetic mechanisms of rust resistance are not well understood in orchardgrass.

**Results:**

In this study, a genome-wide association study (GWAS) was performed using specific-locus amplified fragment sequencing (SLAF-seq) technology in orchardgrass. A total of 2,334,889 SLAF tags were generated to produce 2,309,777 SNPs. ADMIXTURE analysis revealed unstructured subpopulations for 33 accessions, indicating that this orchardgrass population could be used for association analysis. Linkage disequilibrium (LD) analysis revealed an average r^2^ of 0.4 across all SNP pairs, indicating a high extent of LD in these samples. Through GWAS, a total of 4,604 SNPs were found to be significantly (*P* < 0.01) associated with the rust trait. The bulk analysis discovered a number of 5,211 SNPs related to rust trait. Two candidate genes, including cytochrome P450, and prolamin were implicated in disease resistance through prediction of functional genes surrounding each high-quality SNP (*P* < 0.01) associated with rust traits based on GWAS analysis and bulk analysis

**Conclusions:**

The large number of SNPs associated with rust traits and these two candidate genes may provide the basis for further research on rust resistance mechanisms and marker-assisted selection (MAS) for rust-resistant lineages.

**Electronic supplementary material:**

The online version of this article (doi:10.1186/s41065-017-0027-3) contains supplementary material, which is available to authorized users.

## Background

Orchardgrass (*Dactylis glomerata* L.) is a cool-season forage grass that is wildly cultivated in all mild, subtropical regions of the world [1]. Due to its high yield, high nutritional value, and shade tolerance, it has become a valuable pasture species in China [2]. However, orchardgrass is frequently infected by rust disease, which leads to low yield and poor quality [3].

Several studies on rust disease in orchardgrass have been reported. Tajimi et al. [4] studied orchardgrass clonal lines and found that the rusts of orchardgrass, timothy, and ryegrass were closely related and that stem rust was under the genetic control of orchardgrass. Miller and Carlson [5] evaluated rust resistance in orchardgrass based on both phenotypic performance and a polycross progeny test through phenotypic selection. Yan et al. [6] investigated 242 germplasm resources of orchardgrass for 2 years, finding that 13 accessions exhibited high resistance to rust disease. However, the phenotype of an organism can be influenced by both its genotype and its environment. To better assess the impact of this disease, additional methods are needed to study the desirable genetic traits for these plants.

Genome-wide association studies (GWASs) have become one of the most commonly strategies for identifying genes underlying complex traits in plants. In model species, such as *Arabidopsis thaliana*, the potential of GWAS to reveal genetic traits has been demonstrated successfully by the functional validation of the accelerated cell death 6 (ACD6) gene [7]. This approach has also been used to develop a precise estimation of variation in traits such as flowering time [8] of *Arabidopsis thaliana*, leaf architecture [9] of maize (*Zea mays*), and blight resistance [10] of wheat (*Triticum aestivum* L.).In recent studies on rice (*Oryza sativa*), several genes that have large effects on traits involved in determining yield, morphology, and salinity tolerance have been identified [11–13]. In the past few years, flowering time genes in barley (*Hordeum vulgare*) [14], the PsyI-AI locus in wheat [15], and the rhg-1 gene in soybean (*Glycine max*) [16] have been identified as candidate genes [17] through GWAS. Rust disease has seriously influenced plant growth and development, so some studies focus on conducting GWAS to plant rust disease. For example, a GWAS was conducted using 232 winter wheat breeding lines to identify loci conferring resistance to Ug99 that is a new race of stem rust and threatens global wheat production, and 12 loci associated with Ug99 resistance were discovered in this study [18]. A number of 177 oat (*Avena sativa*) accessions were evaluated for disease resistance and further genotyped with 15,000 Diversity Arrays Technology (DArT) and 31 simple sequence repeat and markers to disclose association with disease resistance trait and found five markers were associated with rust resistance [19].

Consequently, several reduced-representation sequencing technologies, including restriction site-associated sequencing (RADseq), double digest RADseq, and two-enzyme genotyping-by-sequencing (GBS), were developed as cost-effective methods for SNP discovery and high-throughput genotyping [20]. Recently, specific-locus amplified fragment sequencing (SLAF-seq), a more efficient solution for large-scale genotyping, was developed by Sun et al. [21]. This approach involves several distinguishing characteristics: (1) fine-mapping resolution among founders can be performed given a high-density of genotypes; (2) outcrossing reshuffles variation in the founder genomes, providing some control over population structure effects; (3) joint-linkage mapping identifies low-resolution quantitative trait loci (QTLs) across all recombinant inbred line (RIL) families, and this genetic background can be controlled while performing nested associations for fine mapping; and (4) the use of RILs allows repeated measurement of phenotypes on the same lines in common and different environments [22]. This approach has previously been used successfully to perform GWAS [23].

SLAF-seq is highly automated due to the development of bioinformatics tools and high-throughput sequencing technology applications. SLAF sequencing methods ascertain the uniformity, efficiency, and density of marker development, and they have been applied in several studies. For example, Li et al. [24] constructed a high-density soybean genetic map to discover QTLs pertaining to isoflavone content that were consistent across various environments; Wei et al. [25] established a high-density SNP map for cucumber (*Cucumis sativus* L.) through SLAF-seq to detect fruit-related QTLs. Additional studies have created high-density genetic maps in order to provide a platform for gene/QTL fine-mapping [20, 26, 27]. These studies showed that SLAF-seq methods are used primarily to construct genetic maps for discovering valuable QTLs; however, for QTL mapping, a large investment of time is needed to establish mapping populations, and the intensive labor required may be a limitation for identification of specific traits. To avoid the limitations of this approach, GWAS is a reliable method used to confirm molecular markers relating to important economic traits. SNP markers that cover the entire genome may be analyzed without the construction of mapping populations [28]. Moreover, several studies have used used SLAF-seq for GWAS [23, 29–31].

In this study, we use the SLAF-seq technology to perform a GWAS of rust traits in orchardgrass to identify the associated SNPs and predict functional genes. These results will provide a basis for orchardgrass breeding and may be helpful in enhancing rust resistance in orchardgrass.

## Results and Discussion

### Sequence and quality statistics

A total of 76.74 M reads were obtained from this experiment. The average Q3 value was 86.91%, and the average GC content was 46.97%. Details are listed in Additional file 1: Table S1.

### Specific-locus amplified fragment sequencing results

In total, 76.74 M reads were generated for the 33 genotypes, encompassing 4.30 Gb of the orchardgrass genomic DNA sequence. A total of 2,334,889 SLAF tags were identified throughout the genome (Table 1). These SLAF tags were divided into three types: Marker (polymorphism tags), No ploy (no polymorphism tags), and Repeat (SLAF tags on repeat sequences) (Table 1). PLINK (v1.07) [32] was used to carry out quality control of the data. Those SNPs with low integrity (<85%), low minor allele frequency (<5%), and insufficient reads (<90%) were discarded. After filtration, 2,309,777 SNPs were used for further analysis.

**Table 1:** The statistical results for each type of SLAF tags

Type	Marker	NoPoly	Repeat	Total
Number	643 008	1 689 409	2 472	2 334 889
Percent	27.54%	72.36%	0.11%	100%

Note: Marker, pleomorphic tags; Nopoly, non-pleomorphic tags; Repeat: SLAF tags on repeat sequence

### Phylogenetic analysis

**Fig. 1:**
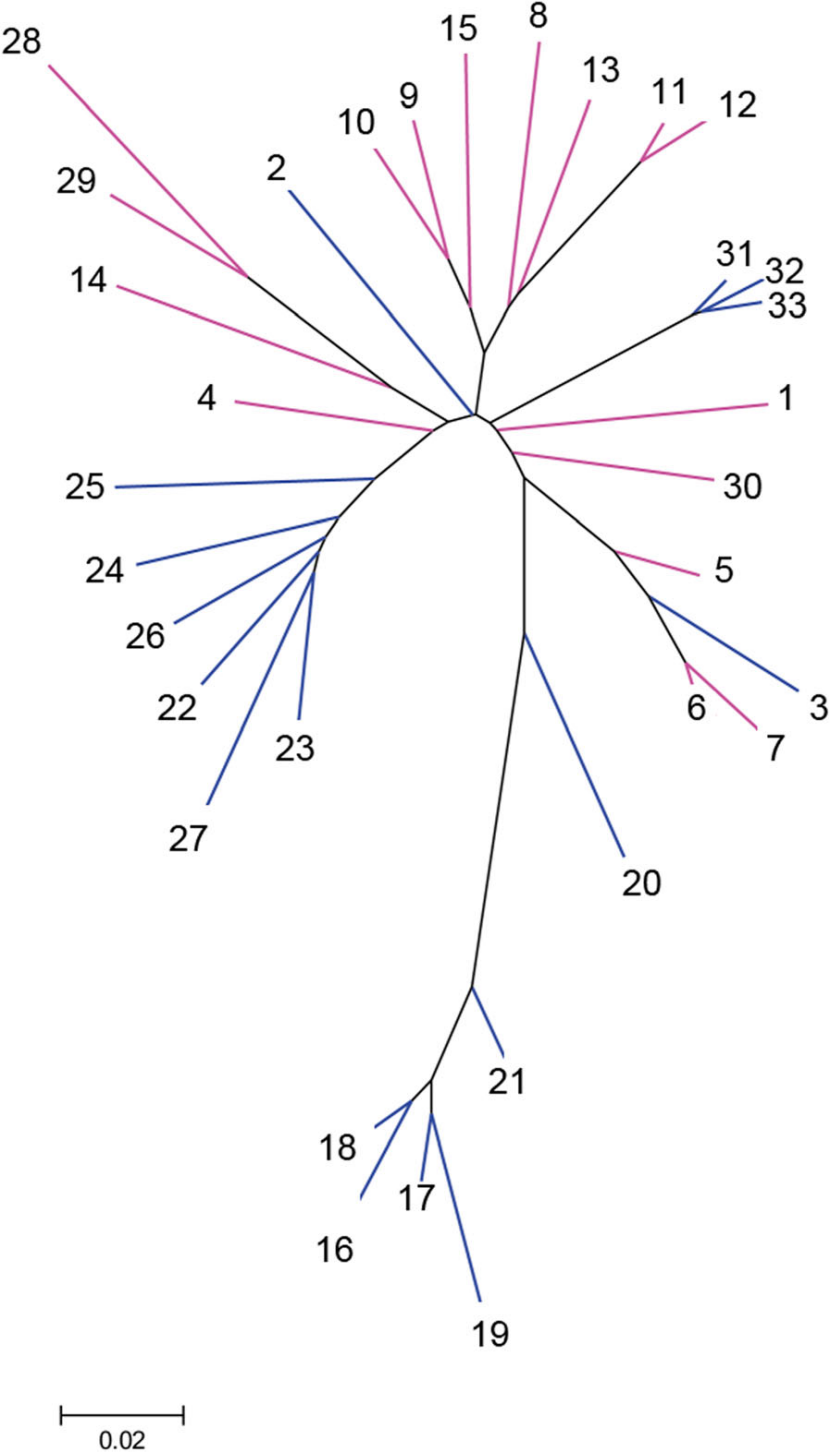
The evolutionary tree of 33 orchardgrass accessions. The branch indicates each material. The red line is HS materials, and blue line is HR materials

The 33 orchardgrass samples could not be clearly divided into subgroups by the neighbor-joining analysis using MEGA software. However, the results from this phylogenetic analysis showed that the HS (high susceptibility of disease) materials were relatively concentrated, comparing with HR (high level of resistance of disease) materials (Fig. 1).

### Group structure and clustering analysis

**Fig. 2:**
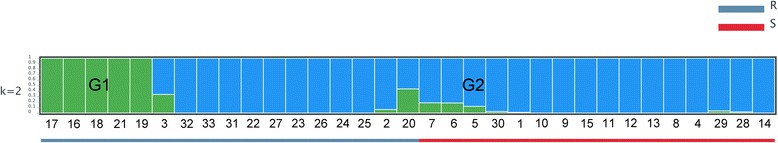
Two subgroups inferred from ADMIXTURE analysis. The vertical coordinated of each subgroup means the membership coefficients for each accession. The digits of the horizontal coordinate represent the accessions according to Table 3. Green zone: G1; Blue zone: G2. The blue line means HR materials; The red line means HS materials

The best dataset was produced by using a K-value of 2, indicating that our samples probably derived from 2 ancestors. With the maximum membership probability, 5 accessions were assigned to group 1 (G1), while 28 accessions were assigned to group 2 (G2). All 5 accessions from G1 were HR samples, and all 16 HS accessions (16) were included in G2 (Fig. 2).

We applied a clustering strategy to the samples with ADMIXTURE software. This method has been used with large sample sizes, exhibiting a strong capability to assign individuals into populations. The best dataset (K = 2) showed that our samples probably derive from two ancestors. It is important to use population-based methods to separate accessions from mixed populations into unstructured subpopulations, allowing for association analyses between phenotypes and molecular bands to be conducted in homogeneous subpopulations [33, 34]. In this study, the accessions associated with resistant and susceptible traits remained in G1, indicating that this orchardgrass population could be used for association analysis.

### PCA analysis

**Fig. 3:**
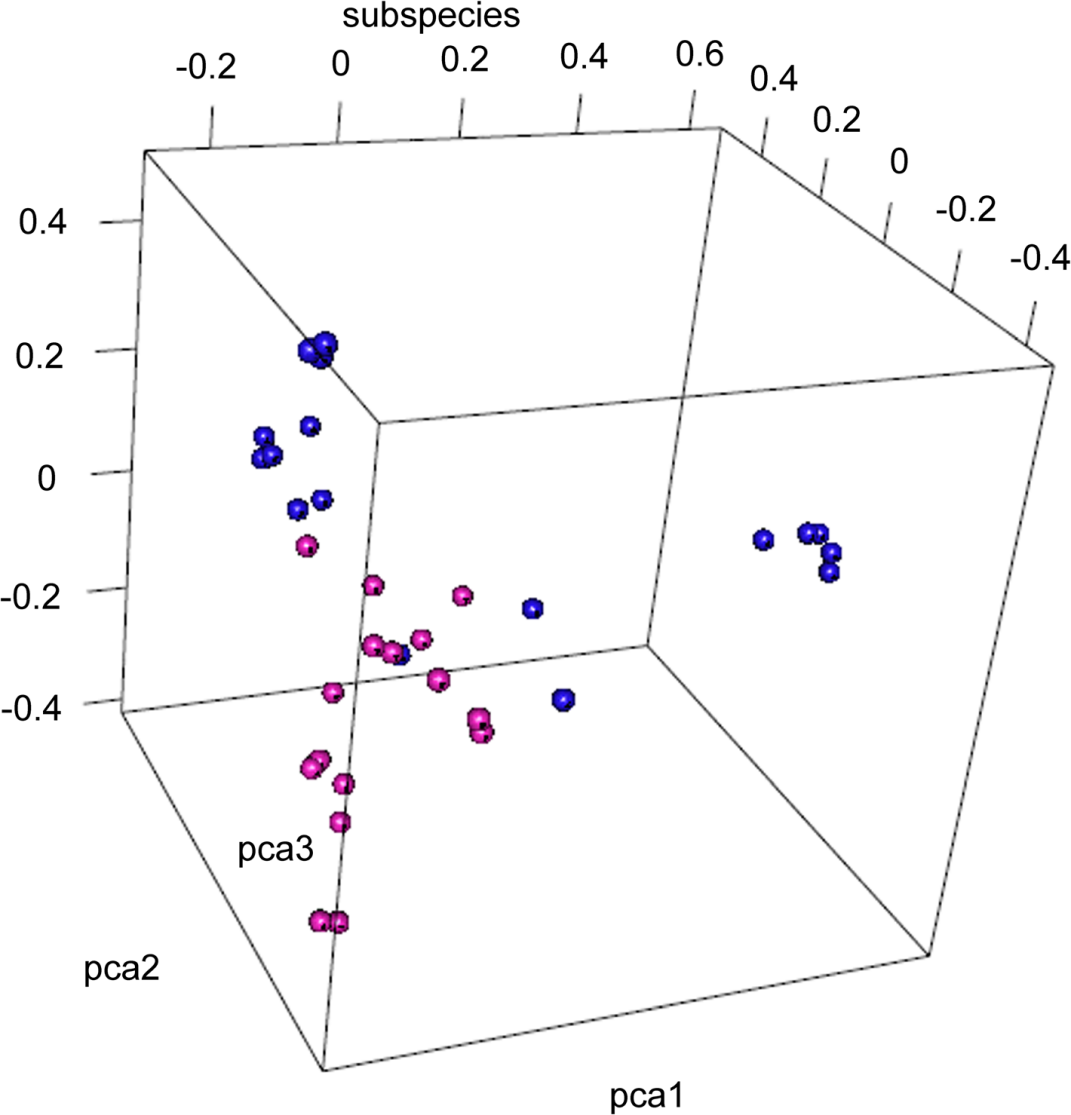
The PCA analysis of 33 orchardgrass accessions. The red balls mean HS materials; the blue balls mean HR materials

A principal component analysis (PCA) was performed using the 2,309,777 SNPs from all 33 accessions to estimate the clusters of population. Principal Component 1 (PC1) explained 7.94% of the variation in the genotypic data, while PC2 and PC3 explained 5.20% and 2.57% of the variation, respectively. Although there are intermediate accessions that make the groupings less clear, the PCA results indicate that the HS samples can be clustered into one group (Fig. 3).

Based on analysis of 2,309,777 SNPs, a neighbor-joining tree was constructed using MEGA software. The results from the phylogenetic analysis showed that the 33 orchardgrass samples could not be clearly divided into two major clusters (Fig. 1), though the 16 HS individuals were able to cluster into one group as shown in the PCA results (Fig. 3). This indicates that there may be no strong relationship between rust resistance and genetic background. Several individuals appeared to be intermediates between the two groups, making the distinction between groups less clear; it may be that we lack a sufficient number of individuals in our sample to appropriately delineate the groups.

### Linkage disequilibrium analysis

The 2,309,777 SNPs generated from the 2,334,889 SLAF markers with unknown chromosome information were used to evaluate the extent of LD in the 33 orchardgrass accessions. In total, 9,345,646 pairs of SNPs were detected and an r^2^ value was calculated for each pair. The values ranged from 0.0000 to 1.0000, with an average r^2^ of 0.4, indicating a substantial amount of LD existing in these SNPs. Of the 9,345,646 pairs, 7,270 (0.08%) were considered to be in LD with strong linkage at r^2^ > 0.8 (Additional file 2).

### GWAS analysis and bulk analysis

The association analysis between SNP markers and the rust trait was performed using TASSEL to search for associated tags and allelic variation. After general linear model (GLM) analysis of 2,309,777 SNPs, 4,604 SNPs were significantly (*P* < 0.01) associated with the rust trait (Additional file 3). At a more stringent P-value cutoff of < 0.001, 1,761 SNPs were still associated with the rust trait (Additional file 4). The bulk analysis was conducted to discover 69,690 SNPs related to the rust trait, and a total number of 5,211 SNPs were filtered when the SNP index is over 0.3 (Additional file 5).

### Bioinformatics analysis of candidate genes

We predicted 555 genes from these 4,604 SNPs in our study (Additional file 6) through GWAS analysis, and only four candidate genes, including cytochrome P450, Pc68LrkC4, prolamin, and EF-hand Ca^2+^-binding protein (TaCab1), were implicated with disease resistance in the Nonredundant NCBI nucleotide sequences (NT) database, and their predicted molecular functions and biological processes are specified in Table 2.

**Table 2:** Bioinformatics analysis of candidate genes

SLAF marker	E_value	Nearest gene	Predict Function
Marker10747	5.00E-18	Cytochrome P450	Resist pathogenic bacterial
Marker11029	2.00E-12	Cytochrome P450	Resist pathogenic bacterial
Marker13729^a^	2.00E-17	Cytochrome P450	Resist pathogenic bacterial
Marker1700^a^	5.00E-13	Cytochrome P450	Resist pathogenic bacterial
Marker3124	5.00E-18	Cytochrome P450	Resist pathogenic bacterial
Marker33680	5.00E-18	Cytochrome P450	Resist pathogenic bacterial
Marker40081	2.00E-11	Cytochrome P450	Resist pathogenic bacterial
Marker4015	2.00E-27	Cytochrome P450	Resist pathogenic bacterial
Marker4728	2.00E-27	Cytochrome P450	Resist pathogenic bacterial
Marker5389	1.00E-08	Cytochrome P450	Resist pathogenic bacterial
Marker12565	6.00E-22	Pc68LrkC4	Activate chemical reactions
Marker2312943	8.00E-16	Pc68LrkC4	Activate chemical reactions
Marker2649	1.00E-24	Prolamin gene	Regulate stripe rust resistance
Marker10408^a^	1.00E-08	Prolamin gene	Regulate stripe rust resistance
Marker21044	8.00E-21	TaCab1	Regulate stripe rust resistance

Note: ^a^means that makers have also been found in bulk analysis

Four genes, including cytochrome P450, Pc68LrkC4, prolamin, and TaCab1, were discovered by detecting genes surrounding each candidate SNP (<1 Mb). Cytochrome P450 plays an important role in the synthesis of secondary metabolites of fungi and metabolism of foreign compounds [35]. Studies on *Populus tomentosa* infected by stripe rust showed that Cytochrome P450 is one of the genes related to germ defense [36]. Pc68LrkC4 was isolated from *Avena sativa* and contains a retrotransposon and repetitive DNA linked to a receptor kinase gene. It has been shown that the leaf rust resistance gene Lr10 of *Triticum aestivum* encodes an extracellular receptor protein kinase with similar domains [37]. Several studies on prolamin have found that proteins with the same or similar rust resistance are closely related to prolamin, and most of these cluster in dendrograms. For example, wheat stripe rust resistance was related to the genetic distance of prolamin, guessing it might because wheat stripe rust gene and partial encoded gene of prolamin were chained on 1BS [38, 39]. In affinity reactions between wheat and stripe rust, the expression of the TaCab1 gene increases significantly. At addition, its expression can be induced by several different biotic stresses. The TaCab1 gene exhibits a marked change in expression after being treated with CaCl2. Researchers have guessed that TaCab1 plays a role in the interaction between wheat and stripe rust via Ca^2+^ transport. In addition, TaCab1 gene silencing increases wheat’s resistance to stripe rust. Therefore, researchers have shown that the TaCab1 gene is correlated with susceptibility to stripe rust [40]. In conclusion, these four candidate genes play important roles in disease progression, particularly the TaCab1 gene. This indicates that these candidate genes are likely to be critical in orchardgrass resistance to rust disease.

We also associated the results between GWAS analysis and bulk analysis to confirm these four predicted genes; however, two types of analysis only shared three markers representing two predicted genes including Cytochrome P450 and Prolamin gene (Table 2). Therefore, these two genes might be more reliable for regulation mechanism of rust resistance in orchgardgrass.

## Method

### Experimental materials

A total of 33 orchardgrass accessions, with high level of resistance of disease (HR) and high susceptibility of disease (HS), as evaluated by Yan et al. [6], were chosen for the experiment, including 17 with HR and 16 with HS in 2011 or 2012 (Table 3). The germplasm of orchardgrass used in this study consisted of 33 accessions that originated from Asia, Africa, Europe, and Oceania. For each accession, 10 individuals were randomly, and 0.5 g of clean young leaves were selected per plant in 2014 for further DNA extraction.

**Table 3:** The detailed information about accession number, name, origin, and rust resistance in orchardgrass for 2011 and 2012

Number	Accession name	Origin	2011	2012
1	01819-6	Beijing,China	HS	HS
2	01824-2	Beijing,China	HR	HR
3	02122-5	Hubei,China	HR	HR
4	231469-1	Libya	HS	HS
5	2410-2	Xinjiang,China	HS	HS
6	2410-6	Xinjiang,China	HS	HS
7	2410-7	Xinjiang,China	HS	HS
8	287804-1	Spain	HS	HS
9	287804-2	Spain	HS	HS
10	287804-3	Spain	HS	HS
11	287804-4	Spain	HS	HS
12	287804-5	Spain	HS	HS
13	287804-8	Spain	HS	HS
14	292587-1	Israel	HS	HS
15	302884-3	Spain	HS	HS
16	308794-1	India	HR	HR
17	308794-2	India	HR	HR
18	308794-3	India	HR	HR
19	308794-5	India	HR	HR
20	308794-7	India	HR	HR
21	308794-8	India	HR	HR
22	325293-2	Russian Federation	HR	HR
23	325293-4	Russian Federation	HR	HR
24	325293-5	Russian Federation	HR	HR
25	325293-6	Russian Federation	HR	HR
26	325293-7	Russian Federation	HR	HR
27	325293-8	Russian Federation	HR	HR
28	578635-7	Morocco	HS	HS
29	578635-8	Morocco	HS	HS
30	79-118-2	Netherlands	HS	HS
31	woronowii(H12)-3	New Zealand	HR	HR
32	woronowii(H12)-4	New Zealand	HR	HR
33	woronowii(H12)-7	New Zealand	HR	HR

Note: *HR* high resistance, *HS* high sense of disease

### Specific-locus amplified fragment sequencing

Total orchardgrass genomic DNA was extracted using the DNeasy Plant Mini Kit (Qiagen USA); the quality and quantity of DNA was then inspected using 0.8% gel electophoresis. The quantified DNA was diluted to 20 μg/μL and was stored at −20 °C before use.

The orchardgrass genomic DNA was analyzed with SLAF-seq [21]. Sequencing libraries of each accession were constructed through digestion with the restriction enzyme HaeIII, (New England Biolabs, USA). A single nucleotide (A) overhang was added to the digested fragments with Klenow Fragment (3′ → 5´ exo–) (New England Biolabs, USA) and dATP at 37 °C, and then duplex tag-labeled sequencing adapters (PAGE purified, Life Technologies, Beijing, China) were ligated to the A-tailed DNA with T4 DNA ligase. The PCR products were purified using Agencourt AMPure XP beads (Beckman Coulter, High Wycombe, UK) and pooled. The pooled sample was separated via electrophoresis in a 2% agarose gel. Fragments with indexes and adaptors from 400 to 450 bp were excised and purified using a QIAquick Gel Extraction Kit (QIAGEN, Duesseldorf, Germany). Finally, the gel-purified product was sequenced using the Illumina HiSeq 2500 system (Illumina, Inc., San Diego, CA, USA). The average depth of sequencing was 6.7×. After sequencing, low-quality reads were filtered out (quality score < 20). Reads with double ends were compared with similar sequences that could be labelled as a candidate SLAF to proceed with the next step. SNPs with low minor allele frequencies (<5%) and low call frequencies (<85%) were deleted [32]. Finally, 33 samples and 2,309,777 SNPs remained for genome-wide association analysis (Additional file 7: Table S2).

### Phylogenetic analysis

Based on the SNP genotype data from the 33 orchardgrass samples, a dendrogram was constructed in MEGA5 [41] using the neighbor-joining algorithm [42]. The structure of the orchardgrass population was analyzed using ADMIXTURE software based on SNP genotype data [43]. The pre-defined K, which indicates the number of groups in a population, varied from 1 to 10 in ADMIXTURE models. A K value was selected when the estimate of lnPr(X|K) peaked in the range of 1 to 10 subpopulations. Delta K (△K), as an ad hoc quantity related to the second order change in the log probability of the data with respect to the number of clusters, was considered as the most probable value of K according to the model choice criteria [42]. A PCA approach with the cluster software [44] was used to cluster the orchardgrass population.

### Evaluation of linkage disequilibrium and GWAS analysis

The squared correlations (r^2^) between all combinations of SNPs (2,309,777) were used to evaluate the significance of pairwise linkage disequilibrium (LD) using Haploview [45] (http://www.softpedia.com/get/Science-CAD/Haploview.shtml). Each pair of SNPs was considered to have strong linkage if r^2^ > 0.8. Based on SNP genotype data, the GWAS analysis was conducted using a GLM in the TASSEL software [43] as follows:

$$ \mathrm{Y}=\mathrm{X}\upalpha +\mathrm{Q}\upbeta +\mathrm{K}\upmu +\mathrm{e}\mathrm{Y} $$

where y is the phenotype value, Q is the population structure matrix calculated by the ADMIXTURE program, X is the genotype matrix, α is the effect of genotype, β is the effect of population structure, μ is the effect of kinship, and e is the residual error. Finally, each SNP locus was assigned a value associated with related traits.

### Bulk analysis

Two groups (HS and HR) were used to conduct a bulk analysis, and the procedures were followed as previously described [46]. To identify potential trait-retaled SNPs, we aligned the short reads obtained from the two DNA bulks to the reference genome (sequences from the HR group were regarded as reference genome) using bwa software [47], and alignment files were converted to SAM/BAM files using SAMtools [48]. SNP-index was calculated for all the SNP positions. We further excluded SNP positions with SNP-index of <0.3 from the two sequences to avoid spurious SNPs called due to alignment errors.

### Bioinformatics analysis of candidate regions

The genetic data relating to rust traits of a 500-Kb window surrounding each SNP were downloaded from Ensembl (http://ensemblgenomes.org/) and NCBI (http://www.ncbi.nlm.nih.gov/). The NT databases were used to conduct pathway analysis and functional annotation for predicted genes [49].

## Conclusions

In this study, SLAF-seq technology was used to conduct the GWAS in orchardgrass, and a number of 2,334,889 SLAF tags were produced to generate 2,309,777 SNPs. The GWAS showed that a total number of 4,604 SNPs were significantly (*P* < 0.01) associated with the rust trait, while a total number of 5,211 SNPs were filtered by bulk analysis. Based on bioinformatics analysis for GWAS results, four candidate genes, including cytochrome P450, Pc68LrkC4, prolamin, and TaCab1 were predicted involving in disease resistance, and the bulk analysis further confirmed cytochrome P450 and prolamin genes were reliable. Besides, further research should be done to determine how these two genes work for resisting diseases. The results can also provide basic information for MAS of rust-resistant lineages.

## Additional files

Additional file 1: (DOCX 21 kb)
Additional file 2: (XLSX 181 kb)
Additional file 3: (XLSX 173 kb)
Additional file 4: (XLSX 79 kb)
Additional file 5: (XLSX 328 kb)
Additional file 6: (XLSX 50 kb)
Additional file 7: (DOCX 19 kb)

## Abbreviations

ACD6: Cell death 6
G1: Group 1
G2: Group 2
GBS: Genotyping-by-sequencing
GLM: General linear model
GWAS: Genome-wide association study
HR: High level of resistance of disease
HS: High susceptibility of disease
LD: Linkage disequilibrium
MAS: Marker-assisted selection
NT: Nucleotide sequences
PC: Principal component analysis
PC1: Principal Component 1
QTLs: Quantitative trait loci
RADseq: Restriction site-associated sequencing
RIL: Recombinant inbred line
SLAF-seq: Specific-locus amplified fragment sequencing
TaCab1: EF-hand Ca^2+^-binding protein
