# Supplementary material for: Genome-wide association study of rust traits in orchardgrass using SLAF-seq technology
Source: Hereditas. 2017 Feb 23;154:5. doi: 10.1186/s41065-017-0027-3 (PMC5322626; doi:10.1186/s41065-017-0027-3)
Supplement: Additional file 1: — (DOCX 21 kb) [file 41065_2017_27_MOESM1_ESM.docx]

Table S1 The sequence data for each orchardgrass accession.

| No | Sample ID | Read Sum | Base Sum | GC (%) | N (%) | Q20 (%) | Cycle Q20 (%) | Q30 (%) |
| --- | --- | --- | --- | --- | --- | --- | --- | --- |
| 1 | 01819-6 | 2406109 | 559226500 | 47.06 | 0.01 | 92.92 | 100 | 87.8 |
| 2 | 01824-2 | 2355038 | 548036855 | 46.6 | 0.01 | 92.63 | 100 | 87.37 |
| 3 | 02122-5 | 2129695 | 497650653 | 46.89 | 0.01 | 93.6 | 100 | 88.71 |
| 4 | 231469-1 | 2578720 | 612449857 | 46.97 | 0.01 | 89.63 | 100 | 82.82 |
| 5 | 2410-2 | 2571204 | 610666735 | 46.7 | 0.01 | 92.9 | 100 | 87.72 |
| 6 | 2410-6 | 2590322 | 613554966 | 46.79 | 0.01 | 92.15 | 100 | 86.74 |
| 7 | 2410-7 | 2116357 | 487274815 | 47.32 | 0.01 | 92.79 | 100 | 87.59 |
| 8 | 287804-1 | 1923136 | 441388198 | 46.9 | 0.01 | 92.77 | 100 | 87.6 |
| 9 | 287804-2 | 2351880 | 546778131 | 46.74 | 0.01 | 93.69 | 100 | 88.87 |
| 10 | 287804-3 | 2553988 | 597539089 | 47.2 | 0.01 | 92.86 | 100 | 87.65 |
| 11 | 287804-4 | 2655645 | 627317902 | 46.79 | 0.01 | 92.92 | 100 | 87.73 |
| 12 | 287804-5 | 2317658 | 537344288 | 47.22 | 0.01 | 92.68 | 100 | 87.34 |
| 13 | 287804-8 | 2177524 | 508270682 | 46.62 | 0.01 | 91.08 | 100 | 84.96 |
| 14 | 292587-1 | 2434339 | 579242528 | 46.35 | 0.01 | 92.5 | 100 | 87.19 |
| 15 | 302884-3 | 1909304 | 429905538 | 47.49 | 0.01 | 93.24 | 100 | 88.3 |
| 16 | 308794-1 | 1858207 | 432450598 | 46.93 | 0.01 | 91.65 | 100 | 85.71 |
| 17 | 308794-2 | 2477912 | 575833514 | 46.95 | 0.01 | 92.95 | 100 | 87.85 |
| 18 | 308794-3 | 2592170 | 623047646 | 46.96 | 0.01 | 89.69 | 100 | 82.98 |
| 19 | 308794-5 | 2943789 | 694529328 | 47.09 | 0.01 | 92.24 | 100 | 86.76 |
| 20 | 308794-7 | 2074008 | 473334476 | 47.55 | 0.01 | 93.14 | 100 | 88.09 |
| 21 | 308794-8 | 2366469 | 560535491 | 46.88 | 0.01 | 90.39 | 100 | 83.89 |
| 22 | 325293-2 | 2351989 | 547297055 | 47.6 | 0.01 | 91.86 | 100 | 86.04 |
| 23 | 325293-4 | 2629612 | 603572663 | 47.33 | 0.01 | 92.87 | 100 | 87.72 |
| 24 | 325293-5 | 2044906 | 471103696 | 47.18 | 0.01 | 92.34 | 100 | 86.91 |
| 25 | 325293-6 | 1949209 | 448992526 | 46.96 | 0.01 | 93.03 | 100 | 87.95 |
| 26 | 325293-7 | 2221131 | 522279842 | 46.74 | 0.01 | 92.67 | 100 | 87.3 |
| 27 | 325293-8 | 2263324 | 525836084 | 47.41 | 0.01 | 92.82 | 100 | 87.65 |
| 28 | 578635-7 | 2064203 | 485166770 | 46.54 | 0.01 | 90.27 | 100 | 83.8 |
| 29 | 578635-8 | 2273594 | 526915818 | 46.98 | 0.01 | 92.9 | 100 | 87.78 |
| 30 | 79-118-2 | 2556417 | 597144309 | 46.44 | 0.01 | 93.31 | 100 | 88.35 |
| 31 | woronowii(H12)-3 | 2388297 | 556303514 | 47.29 | 0.01 | 91.7 | 100 | 86.02 |
| 32 | woronowii(H12)-4 | 2253369 | 523280926 | 46.96 | 0.01 | 92.79 | 100 | 87.53 |
| 33 | woronowii(H12)-7 | 2357951 | 556270252 | 46.61 | 0.01 | 92.58 | 100 | 87.21 |
