# Supplementary material for: Genome-wide association study of rust traits in orchardgrass using SLAF-seq technology
Source: Hereditas. 2017 Feb 23;154:5. doi: 10.1186/s41065-017-0027-3 (PMC5322626; doi:10.1186/s41065-017-0027-3)
Supplement: Additional file 7: — (DOCX 19 kb) [file 41065_2017_27_MOESM7_ESM.docx]

Table S2 The SNP information for each orchardgrass accession.

| Number | Accession name | SNP number | Integrity | Heter ratio |
| --- | --- | --- | --- | --- |
| 1 | 01819-6 | 423 911 | 18.35% | 6.89% |
| 2 | 01824-2 | 391 853 | 16.96% | 6.62% |
| 3 | 02122-5 | 405 889 | 17.57% | 7.19% |
| 4 | 231469-1 | 419 701 | 18.17% | 7.68% |
| 5 | 2410-2 | 452 395 | 19.58% | 7.71% |
| 6 | 2410-6 | 458 769 | 19.86% | 7.86% |
| 7 | 2410-7 | 429 331 | 18.58% | 6.80% |
| 8 | 287804-1 | 389 242 | 16.85% | 6.48% |
| 9 | 287804-2 | 435 206 | 18.84% | 6.72% |
| 10 | 287804-3 | 460 040 | 19.91% | 7.44% |
| 11 | 287804-4 | 444 532 | 19.24% | 7.55% |
| 12 | 287804-5 | 445 443 | 19.28% | 7.01% |
| 13 | 287804-8 | 416 702 | 18.04% | 6.97% |
| 14 | 292587-1 | 367 308 | 15.90% | 8.37% |
| 15 | 302884-3 | 402 041 | 17.40% | 6.06% |
| 16 | 308794-1 | 342 951 | 14.84% | 5.93% |
| 17 | 308794-2 | 403 784 | 17.48% | 6.07% |
| 18 | 308794-3 | 392 420 | 16.98% | 8.58% |
| 19 | 308794-5 | 389 115 | 16.84% | 7.29% |
| 20 | 308794-7 | 399 126 | 17.27% | 5.89% |
| 21 | 308794-8 | 403 949 | 17.48% | 6.97% |
| 22 | 325293-2 | 415 827 | 18.00% | 7.38% |
| 23 | 325293-4 | 470 184 | 20.35% | 7.40% |
| 24 | 325293-5 | 397 202 | 17.19% | 6.54% |
| 25 | 325293-6 | 381 054 | 16.49% | 6.56% |
| 26 | 325293-7 | 408 074 | 17.66% | 6.99% |
| 27 | 325293-8 | 377 440 | 16.34% | 7.25% |
| 28 | 578635-7 | 343 534 | 14.87% | 8.59% |
| 29 | 578635-8 | 383 346 | 16.59% | 7.30% |
| 30 | 79-118-2 | 462 332 | 20.01% | 9.59% |
| 31 | woronowii(H12)-3 | 457 108 | 19.79% | 7.65% |
| 32 | woronowii(H12)-4 | 436 028 | 18.87% | 6.93% |
| 33 | woronowii(H12)-7 | 426 911 | 18.48% | 7.63% |
| Total |  | 2 309 777 |  |  |
